# Percutaneous Soft Tissue Release for Treating Chronic Recurrent Myofascial Pain Associated with Lateral Epicondylitis: 6 Case Studies

**DOI:** 10.1155/2012/142941

**Published:** 2012-12-02

**Authors:** Ming-Ta Lin, Li-Wei Chou, Hsin-Shui Chen, Mu-Jung Kao

**Affiliations:** ^1^Kuan-Ta Rehabilitation and Pain Clinic, Taichung 40652, Taiwan; ^2^Department of Physical Medicine and Rehabilitation, China Medical University Hospital, Taichung 40447, Taiwan; ^3^School of Chinese Medicine, College of Chinese Medicine, China Medical University, Taichung 40402, Taiwan; ^4^Department of Physical Medicine and Rehabilitation, China Medical University, Bei-Gang Hospital, Yun-Lin 65152, Taiwan; ^5^Department of Rehabilitation Medicine, School of Medicine, College of Medicine, China Medical University, Taichung 40402, Taiwan; ^6^Department of Physical Medicine and Rehabilitation, Yangming Branch, Taipei City Hospital, Taipei 11146, Taiwan; ^7^Department of Physical Therapy and Assistive Technology, National Yang-Ming University, Taipei 11221, Taiwan

## Abstract

*Objective*. The purpose of this pilot study is to investigate the effectiveness of the percutaneous soft tissue release for the treatment of recurrent myofascial pain in the forearm due to recurrent lateral epicondylitis. *Methods*. Six patients with chronic recurrent pain in the forearm with myofascial trigger points (MTrPs) due to chronic lateral epicondylitis were treated with percutaneous soft tissue release of Lin's technique. Pain intensity (measured with a numerical pain rating scale), pressure pain threshold (measured with a pressure algometer), and grasping strength (measured with a hand dynamometer) were assessed before, immediately after, and 3 months and 12 months after the treatment. *Results*. For every individual case, the pain intensity was significantly reduced (*P* < 0.01) and the pressure pain threshold and the grasping strength were significantly increased (*P* < 0.01) immediately after the treatment. This significant effectiveness lasts for at least one year. *Conclusions*. It is suggested that percutaneous soft tissue release can be used for treating chronic recurrent lateral epicondylitis to avoid recurrence, if other treatment, such as oral anti-inflammatory medicine, physical therapy, or local steroid injection, cannot control the recurrent pain.

## 1. Introduction

Myofascial pain is a frequent complaint in clinical practice [1–4]. One or more myofascial trigger points (MTrPs) can usually be identified in the muscles responsible for myofascial pain [4]. An MTrP is the most irritable spot in a taut band of skeletal muscle [1, 4], probably due to accumulation of sensitized nociceptors [2, 3]. Almost every normal adult has latent MTrPs, those, which are tender but not painful spontaneously. It becomes active via central sensitization as a consequence of neural or musculoskeletal lesion near or remote to this MTrP [2, 3, 5–7]. An active MTrP is painful spontaneously or in response to movement involving that muscle [4]. An active MTrP can be inactivated after appropriate myofascial pain therapy [4], but recurred frequently if the underlying etiological lesion is not completely removed [2, 3, 6, 8–10]. In clinical practice, an active MTrP can be inactivated immediately after an MTrP injection, but the pain frequently recurs 2-3 weeks after the injection [8, 9]. It appears that the underlying lesion that causes the activation of MTrP is not eliminated [2, 3, 6, 9, 10]. One common example is the pain in the forearm due to MTrPs in the forearm muscles in response to chronic lateral epicondylitis of elbow. 

Lateral epicondylitis (the so-called tennis elbow) is a common elbow pain in clinical practice. It is usually diagnosed in patients with pain over the radial aspect of the elbow, worsened by repetitive or excessive movements of wrist with the elbow in extension, and aggravated by resistive contraction of wrist extensors [11–13]. In addition to the localized pain in the elbow, it can also cause myofascial pain in the wrist and hand extensors [4]. 

The initial management of lateral epicondylitis is conservative [4, 12, 14], with the use of rest, activity modification, nonsteroidal anti-inflammatory drugs, forearm bracing [15], physiotherapy, and local steroid injections [16]. These treatments can provide a transient remission for few months in up to 90% of patients, and 3–8% of patients, who are refractory to conservative treatment, may be surgical candidates [14]. 

Operative management for lateral epicondylitis remains controversial [12]. Since 1922, 14 main surgical treatments modalities with some 300 modifications, have been described [12, 17]. However, it is still unknown whether a given surgical procedure is to be preferred, why each of the different modifications of surgery reports such high success rates, and why some patients fail to respond to surgery [12]. The answer probably lay in the methodology applied in each of these studies [12]. 

Percutaneous release of common extensor tendons at the lateral epicondyle has been used for treating recurrent lateral epicondylitis [18–23]. A sharp surgical knife or an 18G needle (with sharp cut edge) was used for this procedure. 

To avoid excessive tissue damage and bleeding, the first author has developed a new technique by using a cosmetic needle for the release of adhesive soft tissues between the tendon sheath and the periosteum. We have found that this technique can provide successful relief of pain for a significantly long period. This technique is much less invasive comparing to the surgical technique or percutaneous needle release reported previously as mentioned above.

This pilot study is designed to assess the quantitative effectiveness of percutaneous soft tissue release for treating myofascial pain due to lateral epicondylitis.

## 2. Materials and Methods

### 2.1. Patients

Six selected patients with chronic recurrent pain in one elbow and ipsilateral posterior forearm muscles were included in this study. We selected those patients based on the following conditions (standard for this procedure set up by the authors): (1) chronic pain in the lateral epicondyle of one elbow (diagnosed as lateral epicondylitis) and ipsilateral posterior forearm muscles (diagnosed as myofascial pain) for longer than 2 years, (2) treated with physical therapy and oral nonsteroid anti-inflammatory drugs for more than one year with poor results, and (3) treated with local steroid injection with temporary pain relief but recurred within 6 months. We did not include patients with the following conditions: (1) the patient with cognitive deficit, (2) the patient with history of neurological or orthopedic disorder of the involved limb other than pain due to lateral epicondylitis, (3) the patient with any serious medical problem, and (4) the pregnant patient.

The diagnosis of lateral epicondylitis included the following criteria: pain of lateral epicondyle over the radial aspect of the elbow, worsened by repetitive or excessive movements of wrist with the elbow in extension, a tender spot over the lateral epicondyle, and aggravated by resistive contraction of wrist extensors [11–13].

The diagnosis of myofascial pain was based on the exist of MTrPs in one or more muscles in the involved posterior forearm (muscles originate from the common tendon originated from the lateral epicondyle). The criteria for the diagnosis of MTrP included an exquisite tender spot in a palpable taut band of muscle fibers located at the sites indicated in Travell's trigger point manual [1], referred pain or referred tenderness following the patterns described by Travell and Simons [1], and local twitch response in response to the snapping palpation of this spot [1, 4]. 

The Institutional Review Board of the university approved the study and all subjects signed the informed consents for this paper and the assessments with noninvasive routine procedures in the pain clinic. 

The characteristics of these 6 patients are listed in Table 1.

### 2.2. Percutaneous Soft Tissue Release

**Figure 1:**
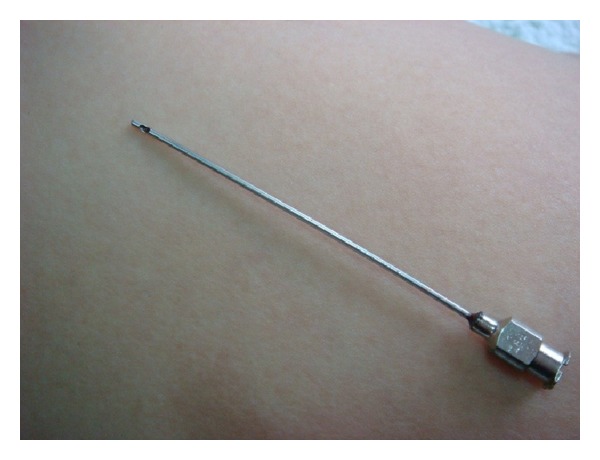
Cosmetic needle used for percutaneous soft tissue release.

Lin [24] has developed a new technique to release the adhesive tissues due to soft tissue lesion by using a blunt cannula (Figure 1). This blunt cannula is originally developed for cosmetic procedure to inject hyaluronic acid into the face or any other tissue. Initially, this procedure had been performed with dry needling. However, the patient developed sore pain for few days after the procedure. Therefore, injection of 1% lidocaine, cortical steroid, and hyaluronic acid was given via a 10 cc syringe connected to the blunt cannula. The addition of local anesthetic was for the immediate relief of pain and also to provide information about the effectiveness of this procedure immediately after treatment. Corticosteroid was used as a strong anti-inflammatory agent. Hyaluronic acid was used for lubrication to avoid readhesion. 

**Figure 2:**
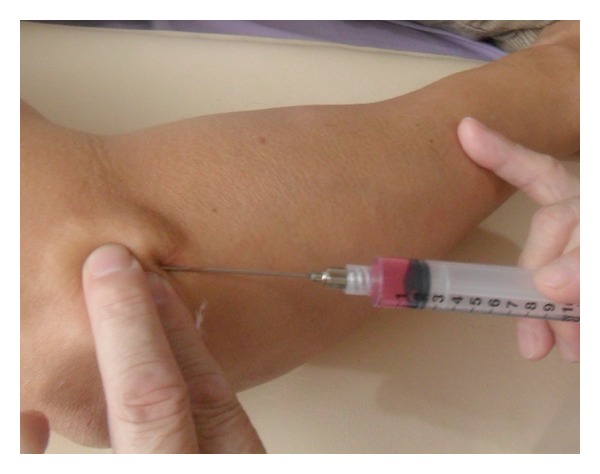
Needle holding for percutaneous soft tissue release.

Initially, the skin around the lateral epicondyle (the origin sites of the common tendons of hand/finger extensors) was cleaned up with povidone-iodine (Betadine). Then, under local anesthesia, the skin was penetrated with an 18 G injection needle to make a hole for the penetration of this blunt cannula. By holding the 10 cc syringe (containing solution as mentioned above) with the dominant hand (Figure 2), the cannula was inserted into the hole to reach the subcutaneous tissue layer, and then moved toward the painful region of the lateral epicondyle slowly. In addition to the forward needle movement, side movement was also performed to release the soft tissues above the common extensor tendons around this track. During needle movement, a drop of solution in the syringe was injected whenever patient complained any pain or discomfort from the needle movement. When the resistance of needle movement was reduced, the needle was pulled back to the subcutaneous layer, and then turned to a different direction for a new track of penetration. Similar to the multiple insertion technique of MTrP injection [9, 25], the blunt cannula was also moved in-and-out to penetrate into different tracks in order to provide a comprehensive release of adhesive soft tissue. Finally, this cannula could sweep around the epicondyle area freely (for an angle about 30 degrees) with no resistance since all adhesive tissues had been released. Then this procedure was completed. 

During this procedure, bleeding up to 10 mL occurred in one case due to injury to a small vein. However, it could be controlled easily immediately after the procedure. In average, the total blood loss during this procedure was less than 3 mL.

### 2.3. Outcome Assessment

**Figure 3:**
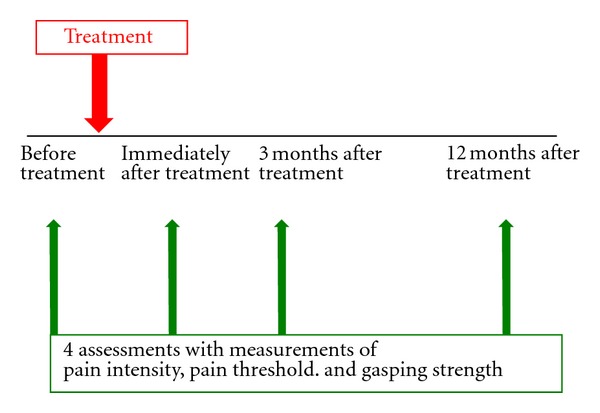
Schedule for outcome assessment.

Assessments of pain intensity, pressure pain threshold, and grasping strength were performed before, immediately after, 3 months after, and 12 months after the needle treatment (Figure 3). 

#### 2.3.1. Subjective Assessment of the Subjective Pain Intensity

The pain intensity over the elbow and forearm of the involved upper limb was assessed based on patient's subjective feeling before, immediately after, and 3 and 12 months after the treatment. It was subjectively reported by the patient using a “Numerical Pain Rating Scale” from zero to ten, with zero (0/10) representing no pain and ten (10/10) representing the worst imaginable pain. The patient was also informed that a value of pain intensity below 5/10 was considered as tolerable pain. 

#### 2.3.2. Assessment of the Pressure Pain Threshold

The pressure pain threshold at tender site of the lateral epicondyle was assessed on every subject before, immediately after, and 3 and 12 months after treatment. The procedure of measurement of the pressure pain threshold recommended by Fischer [26, 27] was applied in this study. The patient was in a comfortable sitting position and was encouraged to maintain complete relaxation. The procedure was explained to the patient clearly. Then the most painful spot in the lateral epicondyle was marked for 3 consecutive measurements so that 3 measurements could be performed over the same area. A pressure algometer (pressure pain threshold meter) was used to measure the pressure pain threshold. This pressure algometer was applied on this marked area with the metal rod perpendicular to the surface of the skin. The pressure of compression was increased gradually at a speed approximately 1 kg/sec. The patient was asked to report any distinct increase of pain or discomfort. The compression stopped as soon as the subject reported that and the reading on the algometer was recorded as a value of pressure pain threshold. The patient was asked to remember this level of pain or discomfort at that point and to apply the same criterion for the next measurement. The patient might demonstrate pain by pulling away or grimacing, which indicated that the pain threshold had been exceeded [26, 27]. If this was the case, the patient was given instructions again and a repeat measurement was taken to ensure that the “real” threshold was obtained. Three repetitive measurements at an interval of 60 seconds were performed at each site. The average values of the three 3 readings (kg/cm^2^) were used for data analysis. One well-trained examiner performed this measurement on all subjects at different times. For the initial assessment, this procedure was performed before and shortly after the needle treatment.

For every patient, the same measurement was performed over the most painful site of lateral epicondyle again 3 months and 12 months after the treatment. Every patient considered the most painful site was consistently the same one at different times. 

#### 2.3.3. Grasping Strength

Grasping strength is primarily measuring finger and hand flexors. However, when the extensors are painful during contraction, such as in the case of tennis elbow, the patient would have weakness in grasping strength since a fixation of wrist is very important to prove a powerful grasping. Ipsilateral hand grasping strength was measured with a hand dynamometer before, immediately after, and 3 and 12 months after treatment. The patient was requested to grasp the dynamometer using the maximal force of finger flexors against the dynamometer with the other end of the hand dynamometer fixed on the base of the palm. Three maximal efforts were tried for each assessment. The average of these 3 force values (kg) was used for data analysis.

### 2.4. Data Analysis

The measured data at different times after needle treatment were compared with the data before treatment based on the analysis of one-way ANOVA. A *P* value less than 0.01 was considered to be statistically significant. 

## 3. Results

### 3.1. Changes in Subjective Pain Intensity

As shown in Table 2, the subjective pain intensity was remarkably reduced in every subject, with further improvement 3 and 12 months after treatment. In the follow-up study one year after the treatment, all subjects reported no pain. The changes in numerical rating scales were statistically significant (*P* < 0.01, Table 2). 

### 3.2. Changes in Pressure Pain Threshold

Table 3 lists the changes in pressure pain threshold over tender spot of the lateral epicondyle before and after therapy. All subjects had remarkably increased pressure pain threshold immediately after therapy. Those effects lasted for up to 12 months. Statistically, those changes were statistically significant (*P* < 0.01, Table 3).

### 3.3. Changes in Grasping Strength

Similar to the improvement in subjective pain intensity and pressure pain threshold, the grasping strength of the involved hand had also been remarkably improved in all subjects, and those effects lasted for up to 12 months. All those changes were statistically significant (*P* < 0.01, Table 4). 

## 4. Discussion

### 4.1. Summary of Important Finding in This Study

This pilot study demonstrated reduced subjective pain intensity, increased pressure pain threshold at the painful site, and increased grasping strength of the involved hand immediately after percutaneous soft tissue release over the lateral epicondylar region of the elbow in treating chronic myofascial pain of the forearm related to lateral epicondylitis. This effectiveness lasted for a period up to one year after treatment.

### 4.2. Correlation of Forearm Myofascial Pain and Lateral Epicondylitis

There have been evidences of the association between active MTrPs and lesions of nonmuscular origins, such as osteoarthritis of knee [28], cervical disc lesion [29], or cervical facet lesion [30]. Chiropractic adjustment [31] or local injection [32] of cervical facet joint could inactivate the MTrPs in the upper trapezius muscles. Bogduk and Simons [30] have suggested the possible connection between facet nociceptors and MTrP nociceptors in the spinal cord and a common use of nociceptive pathway to the higher center from these two kinds of nociceptors. Therefore, when the pain in the facet pain joint is suppressed, the pain due to MTrP can also be controlled, and vice versa. However, in our clinical practice or in searching for the literature, we could not find any case of cervical facet joint pain completely controlled with an MTrP injection of the upper trapezius muscle. On the other hand, facet injection can inactivate the MTrP in the upper trapezius muscle for a long period. Furthermore, if the pain in the upper trapezius MTrP is not elicited by the cervical facet lesion, the pain relief at the MTrP region should not last too long after the facet joint injection. In fact, the long-term relief of an MTrP pain could be observed in this study (longer than one year) and in a previous case report (longer than one year) [33]. Therefore, facet dysfunction may be one of the important causes to activate remote MTrPs. Our current study has further supported the importance of treating the underlying etiological lesion for long-term relief of myofascial pain due to MTrPs [6, 10].

### 4.3. Possible Mechanism of Pain Relief after Percutaneous Soft Tissue Release over the Epicondyle Region

**Figure 4:**
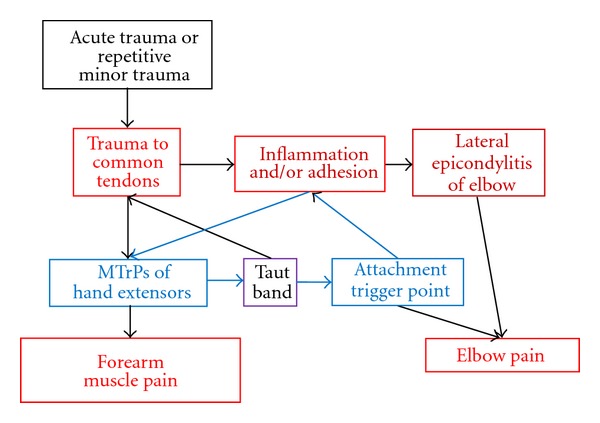
Vicious cycle of elbow and forearm pain.

The adhesion of soft tissues in the lateral epicondyle may be due to fibrosis in chronic inflammation. This chronic inflammation may be caused by direct tendon trauma (either acute pull or chronic repetitive minor trauma). The tendon lesion can activate the MTrP of the hand extensors whose common tendon is coming from the lateral epicondyle [3, 7]. The tendon trauma can be further aggravated by the tension of the taut band related to the MTrP of the hand extensor muscles. This can elicit a vicious cycle of elbow and forearm pain. Furthermore, it is very likely that the adhesion site contains attachment trigger points [4] that can be caused by the chronic tension produced by the taut band of that MTrP. The adhesion in the attachment trigger point region may further activate the MTrP of hand extensors (central sensitization). This condition can elicit another vicious cycle or enhance the whole vicious cycle (Figure 4). Therefore, when the adhesive tissue is released, the whole vicious cycle can be interrupted. Release of adhesive tissues with Lin's technique can provide either direct relief of adhesion or anti-inflammation (injection of local steroid). There the vicious cycle due to either adhesion or inflammation can be interrupted. 

In this study, we also found an immediate relief of pain after the release of soft tissue. Theoretically, the anti-inflammatory effect from local steroid injection is not an immediate process. The immediate pain relief may be related to “hyperstimulation analgesia” from the needle stimulation, similar to MTrP injection or acupuncture [6, 10, 34]. Strong stimuli to nociceptors may elicit strong neural impulses to the spinal cord interneurons, including the hypothetic “MTrP circuit” of an MTrP [6, 10], to inhibit the vicious cycle of pain, and thus provide an immediate pain relief. Therefore, in addition to the adhesion release and anti-inflammatory effect, this procedure may also provide a hyperstimulation analgesic effect. 

However, recent studies have suggested the noninflammatory nature of tendinopathy [35, 36]. It has been considered that lateral epicondylitis of elbow does not involve an inflammatory process of the common extensor origin (CEO). Kraushaar and Nirschl [11] proposed that the pathology is angiofibroblastic hyperplasia of the CEO [37]. Angiofibroblastic hyperplasia can cause soft tissue adhesion and elicit elbow pain. Therefore, surgical tenotomy has been suggested to treat elbow pain due to lateral epicondylitis by excision of the area of angiofibroblastic hyperplasia [12, 17]. Recently, percutaneous release of common extensor tendons at the lateral epicondyle [18–23] has become a popular procedure for treating lateral epicondylitis similar to surgical tenotomy. In fact, Lin's technique of release is one type of tenotomy similar to the procedure performed with percutaneous release of common extensor tendons at the lateral epicondyle [18–23]. However, recent studies have suggested that successful management of tendinopathy does not relate to excision of the actual tendinopathic lesion [38–40]. 

### 4.4. Technique Issues

The open approach of surgical tenotomy can provide a good visualization of the operative field and allows dealing with concomitant pathologies in the elbow [41, 42]. However, it is associated with increased failure rates and complications [41, 43]. It also produces increased time to return to the preinjury level of activity comparing to the procedure of percutaneous techniques [21]. 

The percutaneous technique had a lower complication rate than the open approach of surgical tenotomy [18, 20, 22, 44]. It can be performed as an office procedure. The procedure of Lin's technique is actually a procedure of percutaneous release of adhesion as previously performed by orthopedic surgeon with a knife or a 18 K needle [23]. The major difference between these two procedures is that a blunt cannula instead of a sharp knife or needle is used in this new procedure. Using this new procedure, the recovery period can be much shortened, and the patient has less suffering. 

### 4.5. Limitation of This Study

The major limitation of this study included the small sample sized and the lack of control group. Since this is just a pilot case study, we plan to have further control study on patients of a bigger sample size in the near future. 

## 5. Conclusion

This pilot study indicated therapeutic effectiveness of percutaneous soft tissue release in treating chronic myofascial pain of the forearm related to lateral epicondylitis. Since it is much less invasive than other surgical procedures, this technique can be recommended for the treatment of recurrent lateral epicondylitis with myofascial pain of the forearm muscles with poor responses to conservative treatment (such as oral medicine, physical therapy, or local steroid injection).

## Figures and Tables

**Table 1:** Demographic data of patients.

Case	A	B	C	D	E	F	Mean
Ages (years)	35	48	42	38	53	33	41.5 ± 7.8
Sex	M	F	F	M	M	F	
Side (right/left)	R	R	L	L	R	R	
Duration of pain (years)	3.8	5.2	3.3	2.5	3.1	2.7	3.4 ± 1.0
Trauma history	Sports	None	Traffic accident	None	Sports	None	
Occupation	School teacher	Housewife	Secretary	Constructor	Manager	Housewife	
Previous therapies							
Oral NSAID (months)	12	40	24	30	30	24	26.7 ± 9.3
Physical therapy (months)	18	30	20	12	14	15	18.2 ± 6.5
Local steroid (times)	3	5	3	3	4	2	3.3 ± 1.0
Duration of effectiveness (months)	2-3	3-4	2–4	3–5	3-4	1–3	

**Table 2:** Changes in subjective pain intensity.

Case	Before treatment	Immediately after treatment	3 month after treatment	12 months after treatment
A	8	2	0	0
B	9	1	0	0
C	7	0	1	0
D	8	1	0	0
E	9	2	0	0
F	7	0	0	0

Average	8.0 ± 0.9	1.0 ± 0.9	0.2 ± 0.4	0.0 ± 0.0
*P* value		<0.01	<0.01	<0.01

**Table 3:** Changes in pressure pain threshold (kg/cm^2^).

Case	Before treatment	Immediately after treatment	3 month after treatment	12 months after treatment
A	2.2	3.6	4.0	3.8
B	1.7	3.3	4.2	4.3
C	2.3	2.9	3.9	4.2
D	2.0	2.8	4.1	4.0
E	1.9	3.1	4.2	4.1
F	2.2	3.5	3.3	3.7

Average	2.1 ± 0.2	3.2 ± 0.3	4.0 ± 0.3	4.0 ± 0.2
*P* value		<0.01	<0.01	<0.01

**Table 4:** Changes in strength of hand grasping.

Case	Before treatment	Immediately after treatment	3 month after treatment	12 months after treatment
A	8.1	22.1	27.6	31.2
B	5.3	14.5	18.8	18.4
C	7.8	15.4	22.7	21.0
D	12.7	26.9	27.6	38.1
E	9.3	22.2	31.0	31.2
F	8.8	21.1	23.1	24.2

Average	8.7 ± 2.4	20.4 ± 4.7	25.1 ± 4.4	27.4 ± 7.4
*P* value		<0.01	<0.01	<0.01
